# Are Fish Wild?

**DOI:** 10.1007/s10991-021-09285-0

**Published:** 2021-07-12

**Authors:** Tanya Wyatt, Kim Friedman, Alison Hutchinson

**Affiliations:** 1 Northumbria University, Newcastle, England; 2 The United Nations Food and Agriculture Organization, Fisheries Division, Rome, Italy; 3 The University of Western Australia, Oceans Institute, Crawley, Western Australia Australia

**Keywords:** Fish, Convention on international trade in endangered species of wild fauna and flora (CITES), Wildlife trade, Food security, Animal welfare

## Abstract

As the global biodiversity crisis continues, it is important to examine the legislative protection that is in place for species around the world. Such legislation not only includes environmental or wildlife law, but also trade law, such as the Convention on International Trade in Endangered Species of Wild Fauna and Flora (CITES), which gets transposed into national legislation. This commentary analyses legislative definitions of wildlife, whether or not that includes fish, which has implications for fish welfare, use of fish for food security, and biodiversity conservation when fish, or other wildlife, are excluded. Through a legislative content analysis of the 183 parties’ legislation of CITES, we explore whether fish are afforded the same protections as other species by being included in legal definitions of wildlife. We found that while a majority of CITES parties’ legislation appear to define fish as wildlife, there are a number of instances where this is unclear or not the case, and this could have significant ramifications for the welfare of non-human animals, their use, and conservation.

## Introduction

The global wildlife trade has been thrust into the international discourse in light of the current biodiversity crisis as well as the coronavirus pandemic. As debates surge in regard to whether or not to ban all or part of this lucrative, and many argue necessary, trade, a fundamental component crucial to these debates is largely overlooked—what is wildlife? Whereas intuitively societies and people may have a sense of what wildlife is—non-domesticated animals—there is limited consensus on a definition of wildlife (whether plants and timber are included is another debate (see Wyatt 2021)).

For fish, the situation is confounded by the now contested view that fish do not feel pain or are not sentient (see Browman et al. 2019; Diggles 2019; Franks et al. 2021, among others), and the historical separation of fishing from other forms of hunting. Furthermore, according to Wadewitz (2011), historically, a non-human animal’s physical characteristics and habitat have affected the conservation and management of aquatic versus terrestrial wildlife. In addition, views of what comprises fish have evolved; natural historians of the sixteenth century classified also seals, whales, crocodiles, hippopotamuses and aquatic invertebrates as fish, while the United Nations definition uses a collective term including molluscs, crustaceans, and any aquatic animal which is harvested (FAO 2014; Hickman et al. 2019).

This commentary explores the particular case of whether or not fish are legislatively defined as wildlife and why that is important in the context of global governance of conservation and trade of wildlife as well as international cooperation and law.

## The Relevance of Fish

Fish are the most substantial part of Earth’s animal life, making up almost three quarters of the weight of animals on the planet (Bar-on et al 2018). They are the most consumed non-human animal on the planet, more important than pork and poultry (Brown 2015, FAO 2020) and traded in values exceeding sugar, maize, coffee, rice, and cocoa combined. Despite this importance, global estimates suggest 34.2 percent of capture production is coming from stocks fished at biologically unsustainable levels (FAO 2020), and the status of fish stocks is often poorly assessed, with their management complex due to the dynamic transboundary nature of aquatic systems.

Fish and fish products continue to be the most traded food items in the world today, with most of the world’s countries reporting some fish trade. Over the last several decades, global exports of fish have increased from $15 billion in 1980 to $130 billion in 2020 (FAO 2020); in 2016, about 35 percent of global fish production entered international trade (FAO 2020) and it is estimated that some 78 percent of fish and fish products are exposed to international competition (Tveterås et al 2012). According to the UN Food and Agriculture Organization (FAO 2020: no page), globally, trade in fish and fish products currently represents above “9 percent of total agricultural exports (excluding forest products) and 1 percent of world merchandise trade in value terms”.

Longer and more complex supply chains, enabled by new logistical technologies, a proliferation of multinational corporations pursuing horizontal consolidation and vertical integration, a growing move to culturing fish, and broadening of consumer tastes, concerns, and expectations, makes international cooperation in governance of fish and fish commodities even more important. Cooperation over governance of fish and fish products is essential to the economies of many countries and numerous island, coastal, riverine, insular, and lacustrine regions. For example, fish and fish products exceed 40 percent of the total value of merchandise trade in Cabo Verde, Faroe Islands, Greenland, Iceland, Maldives, Seychelles, and Vanuatu (FAO 2020). In addition to being the most consumed non-human animal, fish are also considered to be one of the most numerous companion animals and one of the most used non-human animals in laboratory research (Brown 2015). Thus, the trade in fish is multifaceted, extensive, and important on many societal and economic levels.

In debates about the governance of trade in wildlife, it is then important to know whether or not this includes fish, and what definition to use to describe fish, which remain vital economic and food staples of many states. In the context of food security, this is important as fish are unique; they are the only ‘wild’ lifeform that provides a large-scale food and nutrient resource from natural environments, with consumption increasing at a rate significantly above that of world population growth (FAO 2020).

Inclusion or not of fish in legal definitions of wildlife is important because of emerging legislation in various parts of the world recognising the sentience of non-human animals and how, therefore, they are allowed to be treated. For instance, in Article 13 of the Treaty on the Functioning of the European Union (EU), fish are recognised as sentient beings (Bauer 2019). However, in further EU welfare legislation fish are minimally mentioned or left out altogether (Bauer 2019). In the current consultations regarding drafting of new environmental and animal welfare legislation in the UK following its departure from the EU, the British Veterinary Association (2020) specifically recommended fish be listed as a recognised sentient non-human animal because of concerns that if they were not specifically mentioned they would be left out of the policy. The importance of legal recognition of sentience in fish is due to the implications such recognition has on legally binding welfare protections. Such protections, which can be linked to whether fish are legally defined as wildlife, could have far-reaching ramifications for the fishing—aquaculture sectors as well as the pet industry and laboratories.

Yet our characterization of fish in global governance frameworks is inconsistent at the most fundamental levels. Possibly most fundamentally, governance of fish in common law jurisdictions, or under the law of torts, characterizes fish as a subset of wildlife only where it is necessary, so that definitions in relation to and interacting with other laws do not raise uncertainty and conflict amongst laws. Yet as a term, the word ‘fish’ is fundamental to characterizing the target of non-human animal welfare protection, food biosecurity, and conservation actions in numerous national and international and regional agreements, where management, control, and protection mechanisms require the cooperation of States.

## Methods

A global instrument for governing trade in wildlife is the Convention on International Trade in Endangered Species of Wild Fauna and Flora (CITES). CITES only governs international trade in the over 38,000 ‘endangered’ species (around 5945 fauna and 32,768 flora) that are listed in its appendices (CITES 2019), but provides useful insight into legal definitions of wildlife, as part of membership requires implementing the Convention into national legislation. Of those listed species, only 154 are fish (CITES 2019) even though as mentioned fish are almost three quarters of the weight of animals on Earth (Bar-on et al 2018). Also, seafood and fish trade are recorded in the greatest numbers within international wildlife trade reporting categories (Anderson et al. 2021), and more than one-third of fish stocks are being captured at biologically unsustainable levels (FAO 2020).

A legislative analysis of all 183 CITES members’ legislation was completed to scrutinize and compare definitions of wildlife (see UK Arts and Humanities Research Council funded study (Wyatt 2021) for related findings). For 112 countries, their legislation is available in English, however there were some limitations to this analysis, i.e., the quality of translation of languages other than Spanish and Russian. Furthermore, CITES parties may have separate fisheries, aquaculture (and welfare) legislation, which was not analysed, but such separation also speaks to the distinction made between fish and other non-human animals.

## What is Wildlife?

**Fig. 1:**
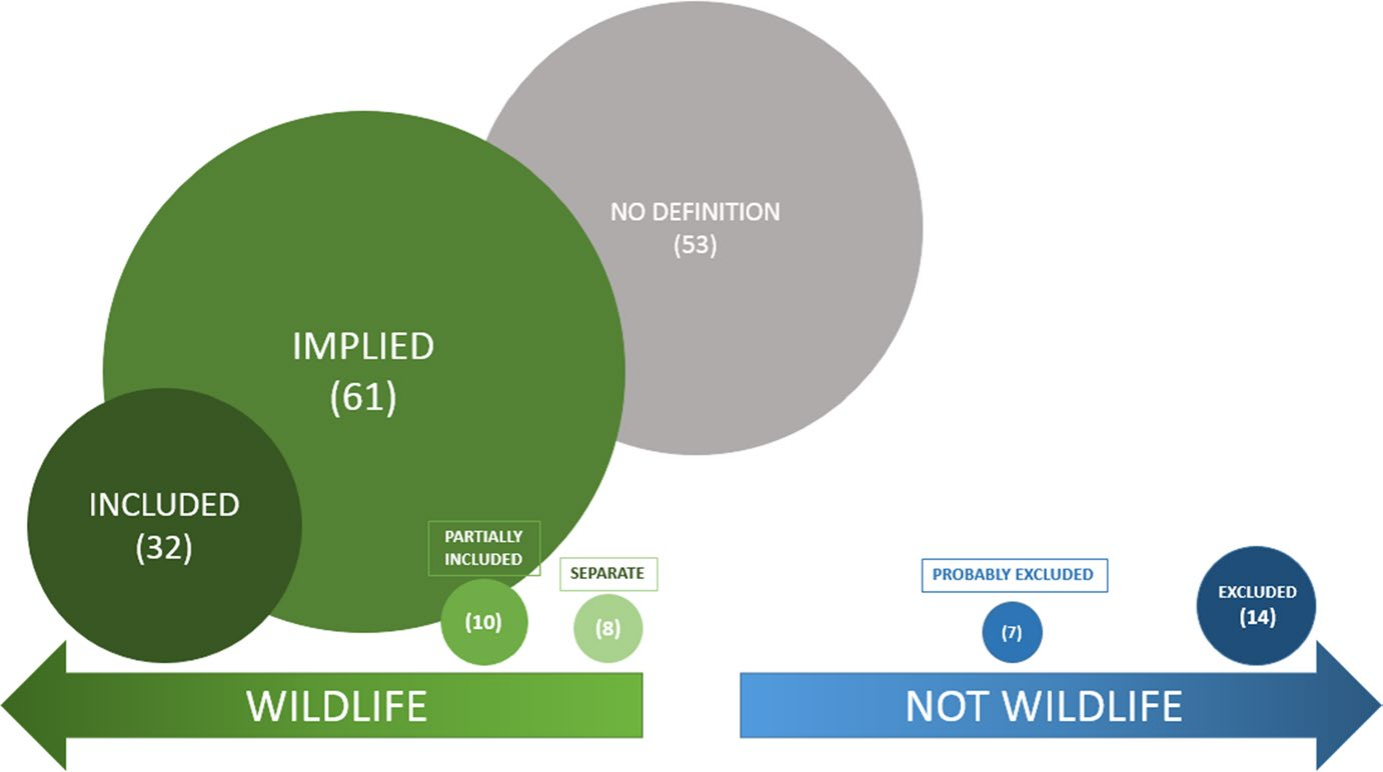
Inclusion or exclusion of fish in CITES transposed legal wildlife definitions (size of the circles infer commonality of use)

Our analysis resulted in seven categories that can be seen along a continuum of fish being fully included as wildlife or fully excluded as wildlife (see Fig. 1). In 32 countries’ legislation, fish are specifically included in definitions of wildlife or animal. For example, in Qatar’s Law No. 5 of 2006 on the Regulation of Trade in Endangered Wildlife Fauna and Flora and their Products, "’Wildlife organisms’ means any member of the animal kingdom including: mammals, birds, fish, amphibians and reptiles, in addition to bacteria or fungus and plants, indigenous or exotic to the natural ecosystem". Conversely, in fourteen instances, fish are explicitly excluded. Mauritius’ legislation—The Native Terrestrial Biodiversity and National Parks Bill No XVI of 2015—states, that “‘wildlife’ includes – (a) any living creature other than –

(i) a human being;
(ii) a dog or cat;
(iii) domestic livestock; or
(iv) fish and other marine organisms”.

‘Partially included’ in definitions of wildlife means that only certain fish were included; in some of these cases, there was a distinction made that river species were included, but no mention was made of marine species. The seven cases where we determined fish are probably not included is in legislation where wildlife or wild animals are specifically listed (i.e., mammals, birds, and reptiles) and no mention of fish are made. The ‘Implied’ category are definitions that say *all* or *any* animals are wildlife and mention regulation of fishing, fisheries, and/or the UN Convention on Law of the Sea. The ‘Separate’ category indicates that the wildlife legislation does not necessarily include fish, but there is indication that another piece of legislation regulates them. Finally, there were 53 instances, where the legislation did not define wildlife or animals.

## The Global Wildlife Trade and Fish

Management and conservation of fish is a global issue that relies on clarity and/or consistency in the use of fundamental terminology. Classification of fishes as wildlife (or not) and their links to governance regimes needs to be clear for global effectiveness of frameworks to conserve the form and function of nature on our planet as well as ensure the welfare of fish. Definitions of wildlife are also important because of the coronavirus pandemic’s links to the wildlife trade. As mentioned, there is an on-going debate about banning wildlife trade and in particular, wildlife ‘wet’ markets. As pointed out in several places (see Alberts 2020; Queen 2020; Standaert 2020 among others), the term wet market has been misused and the discussion should be about markets where live animals of numerous different species intermingle under stressful and unhygienic conditions or killed animals’ bodily fluids mix. Such wildlife markets exist around the world, including in the West, but get called by various names—fishmongers or butchers, for instance. In moving public health debates forward, the discussion of ‘wildlife’ needs to mean the same thing to fisheries and environment government Ministries, trade management authorities, and international institutions and agreements.

Yet, fish often appear to be excluded from wildlife debates. In the case of markets, this may be the case for three reasons. First, as we have demonstrated here, fish are not always considered wildlife. But we are living through a real-time example where the naming of fish—as within or outside of the term wildlife—has important ramifications. Second, although scientific evidence is growing regarding the sentience of fish, the belief that they do not feel pain, so can be captured, and killed in particular ways in large numbers is still accepted, although contested. Third, seemingly, fish are not included in the debates about wildlife markets because they hold less risk to public health as wild fish are generally not a source of zoonotic diseases; however, fish are linked to transmission of parasites and pathogens that can be passed to other aquatic species and people if they enter the food chain (Boylan 2011; Tuševljak et al 2012).

Our findings, albeit from a limited legislative dataset, have significant implications for the relationship between humans and fish. In the CITES context, numerous parties are apparently not completely fulfilling the requirement to regulate trade in listed species because their legal definitions of wildlife do not always include fish, thus excluding CITES-listed fish. Fish not being legally defined as wildlife also has potential negative implications for the sustainability of stocks of non-CITES listed fish species, the governance of wildlife markets, welfare of fish that are held or in trade, and public health measures.

Based upon these findings, it is clear there is a need to examine and reform the legislation of CITES parties that do not include fish, where needed. Closer examination of other legislation governing the trade in wildlife and their welfare is also warranted to ensure fish are not excluded or overlooked, and thus not being protected or posing a risk to public health. This is critical to ensuring the maintenance of the livelihoods of people reliant on fishing — aquaculture industries, to improving fish welfare, and to securing biodiversity and people’s health. To achieve all of this, there needs to be greater clarity, consensus, and inclusivity when defining ‘what is wildlife?’.

The closer examination of wildlife and other relevant legislation would benefit from an interdisciplinary approach. The One Health approach that foregrounds the interface of human —non-human animal — environmental health and the benefits to all from closer cooperation of those working in these fields is a potential model (Amuasi et al 2020; WHO 2017). Fishers, veterinarians, ichthyologists, hobbyists, conservationists, and public health officials among others need to engage in open dialogue analysing the existing legislation and proposing improvements.

This interdisciplinary approach to reforming legislation affecting fish would also need the cooperation of social scientists and legal scholars. Social scientists—criminologists, geographers, political scientists and so forth—are key to designing and implementing programmes, like changes to wildlife trade regulations, where human behaviours are being curtailed. Legal scholars, too, play a role in designing appropriate and non-conflicting legislation, in this case to ensure that all wildlife—fish included—can be jointly protected, managed, and conserved by States across social-environmental value chains. Making sure all wildlife are included in the legislation governing their trade and use is crucial to tackling the biodiversity crisis.

## Data Availability

The datasets compiled for this article and its analysis can be obtained by contacting the authors.

## References

[CR1] Alberts, Elizabeth Claire. 2020. What’s in a name? ‘Wet markets’ may hide true culprits for COVID-19 *Mongabay*. Available at: <https://news.mongabay.com/2020/04/whats-in-a-name-wet-markets-may-hide-true-culprits-for-covid-19/> Accessed 21 April 2021.

[CR2] Amuasi John H, Lucas Tamara, Horton Richard, Winkler Andrea (2020). Reconnecting for our future: The lancet one health commission. The Lancet..

[CR3] Andersson Astrid, Tilley Hannah, Lau Wilson, Dudgeon David, Bonebrake Timothy, Dingle Caroline (2021). CITES and beyond: Illuminating 20 years of global, legal wildlife trade. Global Ecology and Conservation..

[CR4] Bar-on Yinon, Phillips Rob, Milo Ron (2018). The biomass distribution on Earth. PNAS.

[CR5] Bauer Helena (2019). Fishes: The forgotten sentient beings. Derecho Animal (forum for Animal Law Studies)..

[CR6] Boylan Shane (2011). Zoonoses Associated With Fish. Veterinary Clinic of North America: Exotic Animal Practice.

[CR7] British Veterinary Association (BVA). 2020. Recognition of Sentience. *The Veterinary Record* 187(12): 506-506. 10.1136/vr.m4895.

[CR8] Browman Howard, Cooke Steven, Cow Ian, Derbyshire Stuart, Kasumyan Alexander, Key Brian, Rose James, Schwab Alexander, Anne Skiftesvik E, Stevens Don, Watson Craig, Arlinghaus Robert (2019). Welfare of aquatic animals: Where things are, where they are going, and what it means for research, aquaculture, recreational angling, and commercial fishing. ICES Journal of Marine Science..

[CR9] Brown Culum (2015). Fish intelligence, sentience and ethics. Animal Cognition..

[CR10] Convention on International Trade in Endangered Species of Wildlife Fauna and Flora (CITES). 2019. The CITES Species. Available at: <https://www.cites.org/eng/disc/species.php> Accessed 21 April 2021.

[CR11] Diggles BK (2019). Review of some scientific issues related to crustacean welfare. ICES Journal of Marine Science..

[CR12] Food and Agricultural Organization (FAO). 2014. FAO Fisheries Glossary. Available at: <http://www.fao.org/faoterm/collection/fisheries/en> Accessed 21 April 2021.

[CR13] Food and Agricultural Organization (FAO). 2020. The State of World Fisheries and Aquaculture 2020 Sustainability in action. Available at: <10.4060/ca9229en> Accessed 21 April 2021.

[CR14] Franks Becca, Ewell Christopher, Jacquet Jennifer (2021). Animal welfare risks of global aquaculture. Science Advances.

[CR15] Hickman Cleveland, Keen Susan, Eisenhour David, Larson Allan, I’Anson Helen (2019). Integrated principles of zoology.

[CR16] Queen, Green. 2020. Stop confusing wet markets with wildlife markets. *Africa Sustainable Conservation News*. Available at: <https://africasustainableconservation.com/2020/04/21/stop-confusing-wet-markets-with-wildlife-markets/> Accessed 21 April 2021.

[CR17] Standaert, Michael. 2020. ‘Mixed with prejudice’: calls for ban on ‘wet’ markets misguided, experts argue. *The Guardian*. Available at <https://www.theguardian.com/environment/2020/apr/15/mixed-with-prejudice-calls-for-ban-on-wet-markets-misguided-experts-argue-coronavirus> Accessed 21 April 2021.

[CR18] Tuševljak Nataša, Rajić Andrijana, Waddell Lisa, Dutil Lucie, Cernicchiaro Natalia, Greig Judy, Wilhelm Barbara J, Wilkins Wendy, Sarah Totton F, Uhland Carl, Avery Brent, McEwen Scott A (2012). Prevalence of zoonotic bacteria in wild and farmed aquatic species and seafood: A scoping study, systematic review and meta-analysis of published research. Foodborne Pathogens and Disease..

[CR19] Tveterås Sigbjørn, Asche Frank, Bellemare Marc F, Smith Martin D, Guttormsen Atle G, Lem Audun, Lien Kristin, Vannuccini Stefania (2012). Fish is food - The FAO’s fish price index. PLoS ONE.

[CR20] Wadewitz Lissa (2011). Are fish wildlife?. Environmental History..

[CR21] World Health Organization (WHO). 2017. One Health. Available at: <https://www.who.int/news-room/q-a-detail/one-health> Accessed 21 April 2021.

[CR22] Wyatt T (2021). Is CITES protecting wildlife? Assessing implementation and compliance.

